# Targeting N-Terminal Human Maltase-Glucoamylase to Unravel Possible Inhibitors Using Molecular Docking, Molecular Dynamics Simulations, and Adaptive Steered Molecular Dynamics Simulations

**DOI:** 10.3389/fchem.2021.711242

**Published:** 2021-08-30

**Authors:** Shitao Zhang, Yi Wang, Lu Han, Xueqi Fu, Song Wang, Wannan Li, Weiwei Han

**Affiliations:** ^1^ School of Life Science, Key Laboratory for Molecular Enzymology and Engineering of Ministry of Education, National Engineering Laboratory for AIDS Vaccine, Jilin University, Changchun, China; ^2^ Laboratory of Theoretical and Computational Chemistry, Institute of Theoretical Chemistry, Jilin University, Changchun, China

**Keywords:** maltase-glucoamylase, inhibitors, molecular dynamics simulations, adaptive steered molecular dynamics simulations, conformational change

## Abstract

There are multiple drugs for the treatment of type 2 diabetes, including traditional sulfonylureas biguanides, glinides, thiazolidinediones, α-glucosidase inhibitors, glucagon-like peptide-1 (GLP-1) receptor agonists, dipeptidyl peptidase IV (DPP-4) inhibitors, and sodium-glucose cotransporter 2 (SGLT2) inhibitors. α-Glucosidase inhibitors have been used to control postprandial glucose levels caused by type 2 diabetes since 1990. α-Glucosidases are rather crucial in the human metabolic system and are principally found in families 13 and 31. Maltase-glucoamylase (MGAM) belongs to glycoside hydrolase family 31. The main function of MGAM is to digest terminal starch products left after the enzymatic action of α-amylase; hence, MGAM becomes an efficient drug target for insulin resistance. In order to explore the conformational changes in the active pocket and unbinding pathway for NtMGAM, molecular dynamics (MD) simulations and adaptive steered molecular dynamics (ASMD) simulations were performed for two NtMGAM-inhibitor [de-O-sulfonated kotalanol (DSK) and acarbose] complexes. MD simulations indicated that DSK bound to NtMGAM may influence two domains (inserted loop 1 and inserted loop 2) by interfering with the spiralization of residue 497–499. The flexibility of inserted loop 1 and inserted loop 2 can influence the volume of the active pocket of NtMGAM, which can affect the binding progress for DSK to NtMGAM. ASMD simulations showed that compared to acarbose, DSK escaped from NtMGAM easily with lower energy. Asp542 is an important residue on the bottleneck of the active pocket of NtMGAM and could generate hydrogen bonds with DSK continuously. Our theoretical results may provide some useful clues for designing new α-glucosidase inhibitors to treat type 2 diabetes.

## Introduction

At present, there are multiple drugs for the treatment of type 2 diabetes, including traditional sulfonylureas (Stephen et al., 2018), biguanides (Schäfer, 1983), glinides (Chen et al., 2015), thiazolidinediones (Nanjan et al., 2018), α-glucosidase inhibitors (Kazufumi et al., 2014; Patel, 2015), glucagon-like peptide-1 (GLP-1) receptor agonists (Drucker, 2018), dipeptidyl peptidase IV (DPP-4) inhibitors, and sodium-glucose cotransporter 2 (SGLT2) inhibitors (Thornberry and Gallwitz, 2009; Kelly et al., 2019). These therapeutic drugs have been widely used in clinical trials because of their own characteristics in hypoglycemic control. For example, α-glucosidase inhibitors have been used to control postprandial glucose levels caused by type 2 diabetes since 1990 (Ríos et al., 2015; Flores-Bocanegra et al., 2017; Santos et al., 2018; Dhameja and Gupta, 2019; Usman et al., 2019; Mi et al., 2021; Tuyen et al., 2021). Acarbose (Chiasson et al., 2002) and miglitol (Satoru et al., 2015), which were clinically used for treating type 2 diabetes, may control blood glucose levels by targeting α-amylases and α-glucosidases (Lyann et al., 2010; Ren et al., 2011).

Glycoside hydrolases play significant roles in human metabolism, including digestion and decomposition of polysaccharides and biosynthesis of glycoprotein (Lovering et al., 2005). α-Glucosidases are rather crucial in the human metabolic system and are principally found in families 13 and 31 (Lovering et al., 2005). Maltase-glucoamylase (MGAM) (Sim et al., 2008) and sucrase-isomaltase (SI) (Sim et al., 2010) belong to glycoside hydrolase family 31. The main function of MGAM and SI is to digest terminal starch products left after the enzymatic action of α-amylase, which becomes an efficient drug target for insulin resistance (Van Beers et al., 1995). MGAM contains the following units: a small cytosolic domain of approximately 26 residues, a transmembrane domain (TMD) containing about 20 residues inserted into the intestinal epithelial cell membrane, an O-glycosylated linker, and two independent catalytic subunits: NtMGAM and C-terminal luminal subunit (CtMGAM) (Sim et al., 2008; Lyann et al., 2010) (Supplementary Figure S1). NtMGAM containing 864 residues (PDB ID: 3L4U) (Lyann et al., 2010) were used in this study. NtMGAM, known to be retaining α-glycosidases (Satoh et al., 2016), has received relatively little attention despite its importance and the number of different activities from a range of organisms, including mammals, plants, and microorganisms (Frandsen and Svensson, 1998; Yu et al., 1999; Lovering et al., 2005). The substrate specificities of MGAM vary and overlap from maltose (Quezada-Calvillo et al., 2008) to isomaltose (Elferink et al., 2020) and other small oligosaccharides.

*Salacia reticulata (S. reticulata)*, a plant widely distributed in China and some other countries in Southeast Asia, is used as a traditional prescription for treating type 2 diabetes (Medagama, 2015). Sulfonium ion-containing compounds were isolated from aqueous extracts of *S. reticulata* by Jayakanthan and his colleagues (Jayakanthan et al., 2009), including de-O-sulfonated kotalanol (DSK). DSK comprises a 1,4-anhydro-4-thio-d-arabinitol core and a polyhydroxylated acyclic chain (Lyann et al., 2010) and can act as an inhibitor of α-glucosidase in human bodies. A previous study reported that DSK could be an efficient inhibitor of NtMGAM with a K_i_ of 0.03 (±0.01) μM (Lyann et al., 2010).

In order to explore the conformational changes in the active pocket and unbinding pathway for NtMGAM, molecular dynamics (MD) simulations (Zhu et al., 2018; Zhu et al., 2019; Liu et al., 2020) and adaptive steered molecular dynamics (ASMD) simulations (Zhu et al., 2018) were performed between two inhibitors (DSK and acarbose) and NtMGAM (PDB ID: 3L4U) (Lyann et al., 2010) (workflow listed in Supplementary Figure S2). Our results may provide new ideas for the further design of α-glucosidase inhibitors.

## Materials and Methods

### Preparation for the Structure of Protein Inhibitors

AutoDock 4.2 (Morris et al., 2009) was used for docking acarbose with NtMGAM using the Lamarckian genetic algorithm to identify a proper binding conformation with a grid box size of 66 Å × 58 Å × 66 Å points and a grid point spacing of 0.375 Å. The binding conformation with the lowest energy was chosen for simulations. The crystal structure of NtMGAM with DSK complex and the 3D structure of acarbose (PDB ID: 3JYR) (Vahedi-Faridi et al., 2010) was downloaded from Protein Data Bank (www.rcsb.org) for further studies.

### MD Simulations

Simulations in our study were performed using the Amber16 package (D.A. Case et al., 2016) with the Amber ff99SB force field (Lindorff-Larsen et al., 2010). At the same time, the Gaff2 force field (Wang et al., 2004) was utilized to generate the parameterization of DSK and acarbose. It is well known that charged residues affect the environment of protein (Popović and Stuchebrukhov, 2004; Tashiro and Stuchebrukhov, 2005; Sugitani and Stuchebrukhov, 2009). H++ is an online tool that can automatically compute pKa values of ionizable groups in proteins (http://biophysics.cs.vt.edu/). We computed the ionizable groups of NtMGAM on H++ and then manually fixed the ionizable groups. All three complexes were analyzed using the MD simulations in a cubic periodic boundary box with the TIP3P water model (Bogunia and Makowski, 2020), which was prolonged to 12 Å from the protein atoms. Sodium ions were randomly added to the simulation systems for neutralization. To get the initial equilibrious structure, energy minimization was performed through the steepest descent method in 1,000 cycles. Subsequently, 50 ps of NVT (Berendsen temperature coupled with constant particle number, volume, and temperature) (Berendsen et al., 1984) and 50 ps of NPT (Parrinello–Rahman pressure coupled with constant particle number, pressure, and temperature) (Andersen, 1980) were performed to maintain the stability of the system (300 K, 1 bar). After stabilizing all thermodynamic properties, a 200 ns unconstrained MD simulation was performed with a time interval of 2 fs. The coordinates for all models were stored every 2 ps. During the simulation, the following options were specified: (I) bonds involving hydrogen are constrained and bond interactions involving H-atoms were omitted using the SHAKE algorithm (Miyamoto and Kollman, 1992), (II) the particle mesh Ewald summation algorithm (Essmann et al., 1995) was taken to calculate the long-range electrostatic interactions, (III) 1 atm constant pressure was maintained by the Langevin dynamics method (Rosenberg et al., 1986) (Guàrdia and Padró, 1985), and (IV) an optimum temperature (300 K) was maintained. MD simulations were performed three times for each system in this study (Supplementary Figures S3, S4). The root-mean-square deviation (RMSD), radius of gyration (R_g_), root-mean-square fluctuation (RMSF), and solvent-accessible surface area (SASA) values were calculated using VMD (Humphrey et al., 1996).

### MM-PBSA Calculations

MMPBSA.py (Miller et al., 2012) in the AmberTools17 package was employed to conduct free energy calculations for the two complexes. 200 conformations were extracted from each equilibrious trajectory (from 100 to 200 ns with an interval of 50 frames) for calculations. The binding free energies were calculated by subtracting the free energies of the receptor and the ligand DSK or acarbose from the free energy of the bound complex of two systems:

$$\Delta G_{binding,solvated}=\Delta G_{complex,solvated}-\left[\Delta G_{receptor,solvated}+\Delta G_{ligand,solvated}\right].$$

Then, the free energy change associated with each term of ΔG was calculated according to the following:

$$\Delta G_{solvated}=E_{gas}+\Delta G_{solvation}-TS_{solute},$$

where *ΔG*_*solvation*_ represents true free energy. To determine the relative stability, end-state method calculations were performed to estimate the energies, according to averages from the ensemble of these snapshots:

$$\Delta G_{solvated}≅<E_{gas}>+<\Delta G_{solvation}>-T<S_{solute}>=\frac{1}{N}\overset{N}{\underset{i=1}{\sum }}E_{i,gas}+\frac{1}{N}\overset{N}{\underset{i=1}{\sum }}\Delta G_{i,solvation}-\frac{T}{N}\overset{N}{\underset{i=1}{\sum }}S_{i,solute},$$

where *i* is the index of a particular frame and N is the total number of frames analyzed. The gas-phase energies (E_gas_) can be computed from the quantum mechanical (QM) calculations and used further as a part of the force field parameterization; therefore, the E_gas_ energies can be abstracted from the molecular mechanical (MM) energies and the corresponding force field (Miller et al., 2012).

### Principal Component Analysis and Free Energy Landscapes

PCA is a common statistical multivariate method, which can select the structure of each frame in an MD trajectory as a new set of variables, called principal components (PCs), with a minimal loss of information. In our study, we employed the bio3d package in R to refine structural superposition and examine the relationship between different conformers (Grant et al., 2006). The current protocol excludes the residues displaying the largest positional differences at each round and identifies only the core residues. Following the superposition of core residues, PCA was performed to examine the conformers based on their equivalent residues. The PCs collected during MD simulation are originally the eigenvector values collected from the covariance matrix, each corresponding to the change in protein trajectory (Al-Khafaji and Taskin Tok, 2020). In order to obtain a lower-dimensional representation of the structural dataset, we project the distribution onto the subspace defined by the largest principal components. In each dimension, the corresponding eigenvalue represents the percentage of the total mean square displacement (or variance) of atom positional fluctuations. In PCA, very few dimensions are generally enough to capture about 70% of the total variance in the structures to be studied (Grant et al., 2006). Therefore, the first few eigenvectors are sufficient to provide a useful description while still retaining most of the variance in the original distribution. After PCA, the FEL (Frauenfelder et al., 1991) was obtained by calculating the joint probability distribution from the essential plane constructed from the top two eigenvectors (Singh et al., 2015).

### Pathways Identified With CAVER

The software called CAVER has been widely used to analyze and visualize possible cavities and channels in protein structures. CAVER Analyst 2.0 (Jurcik et al., 2018) was employed to determine the channel position. The starting point was set at the position of the ligand for the channel computation. The minimum probe radius, clustering threshold, shell depth, and radius were set to be 0.9, 3.5, 4, and 3 Å, respectively. All the other parameters were used as default. 1,000 snapshots (we took one frame every ten frames in 10,000 from the 100–200 ns simulations) were analyzed utilizing CAVER Analyst 2.0.

### Adaptive Steered Molecular Dynamics Simulations

ASMD simulations (Ozer et al., 2010; Ozer et al., 2012a; Ozer et al., 2012b; Ozer et al., 2014) were performed for NtMGAM with the two different ligands using the Amber 16 package. ASMD has been shown to alleviate the problem that many simulations must be run to converge the potential mean of force (PMF) in steered molecular dynamics (Izrailev et al., 1999) by dividing the predetermined reaction coordinate into numerous smaller stages. Each stage in the ASMD simulation contained multiple simulations that should be performed parallelly. In each stage, the trajectory with the work value closest to the Jarzynski average (JA) (Jarzynski, 1997) should be selected, and the coordinates at the end of that trajectory should be used as an initial coordinate for the next stage. Then, the JA structures were used for PMF calculation. Here, in our study, the distance between the ligand and NtMGAM in the initial conformation is 6 Å. The reaction coordinate in ASMD is predetermined to be set at 20 Å, at which the inhibitor is considered to escape from the enzyme. The stretching velocity was 10 Å/ns in this ASMD simulation, coupling a spring constant k of 40 kcal/(mol×Å^2^). Each simulation was split every 2 Å into seven stages; each stage contains 14 simulations to reach the final reaction coordinate of 20 Å. As the distance between the two selected atoms reached 20 Å, there were no longer any interactions between the ligand and the receptor.

## Results and Discussion

### Docking Pose and System Stabilize

It was reported that the structure of the NtMGAM substrate-binding site consisted of two sugar-binding sites, which in the acarbose-NtMGAM binding structure were occupied by the two nonreducing rings of acarbose (Lyann et al., 2010). To determine the docking pose, we chose to dock the DSK crystal structure to NtMGAM with AutoDock 4.2 (Morris et al., 2009). Comparing the crystal structure of DSK-NtMGAM and the docked pose (Supplementary Figure S5), it can be concluded that they were similar (RMSD 0.50), which indicated that this system may use AutoDock 4.2 software to determine the binding pose. Acarbose was docked to NtMGAM using the same method.

**FIGURE 1:**
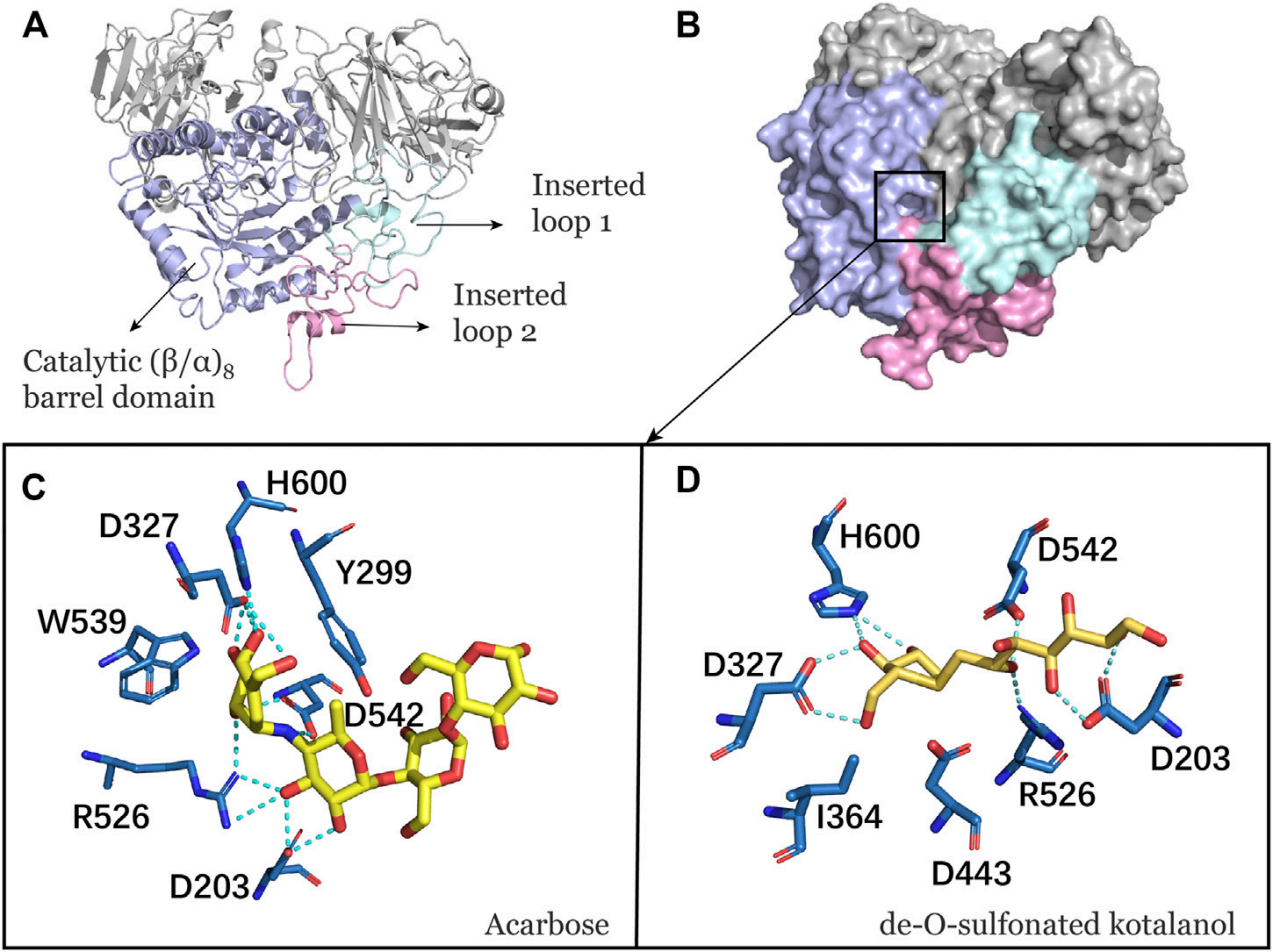
**(A)** Catalytic domain of NtMGAM. The catalytic (β/α)_8_ barrel domain is tagged and colored differently. **(B)** Surface diagram of NtMGAM. **(C)** Binding of acarbose to NtMGAM. The active residues around acarbose binding to NtMGAM. **(D)** Binding of DSK to NtMGAM. The active residues around DSK binding to NtMGAM.

NtMGAM has a typical catalytic (β/α)_8_ barrel domain (Figure 1A). DSK and acarbose were docked in an active pocket of NtMGAM (Figure 1B). Figures 1C,D shows that the active residues around acarbose and DSK were bound around NtMGAM. It can be seen that His600, Arg526, Asp542, Asp203, Trp406, and Asp327 were anchor residues for DSK binding in Figure 1D. In contrast, in the acarbose-NtMGAM, His594, Asp321, Arg520, Asp197, and Asp536 made hydrogen bonds with acarbose, indicating that they are important residues for acarbose binding to NtMGAM.

**FIGURE 2:**
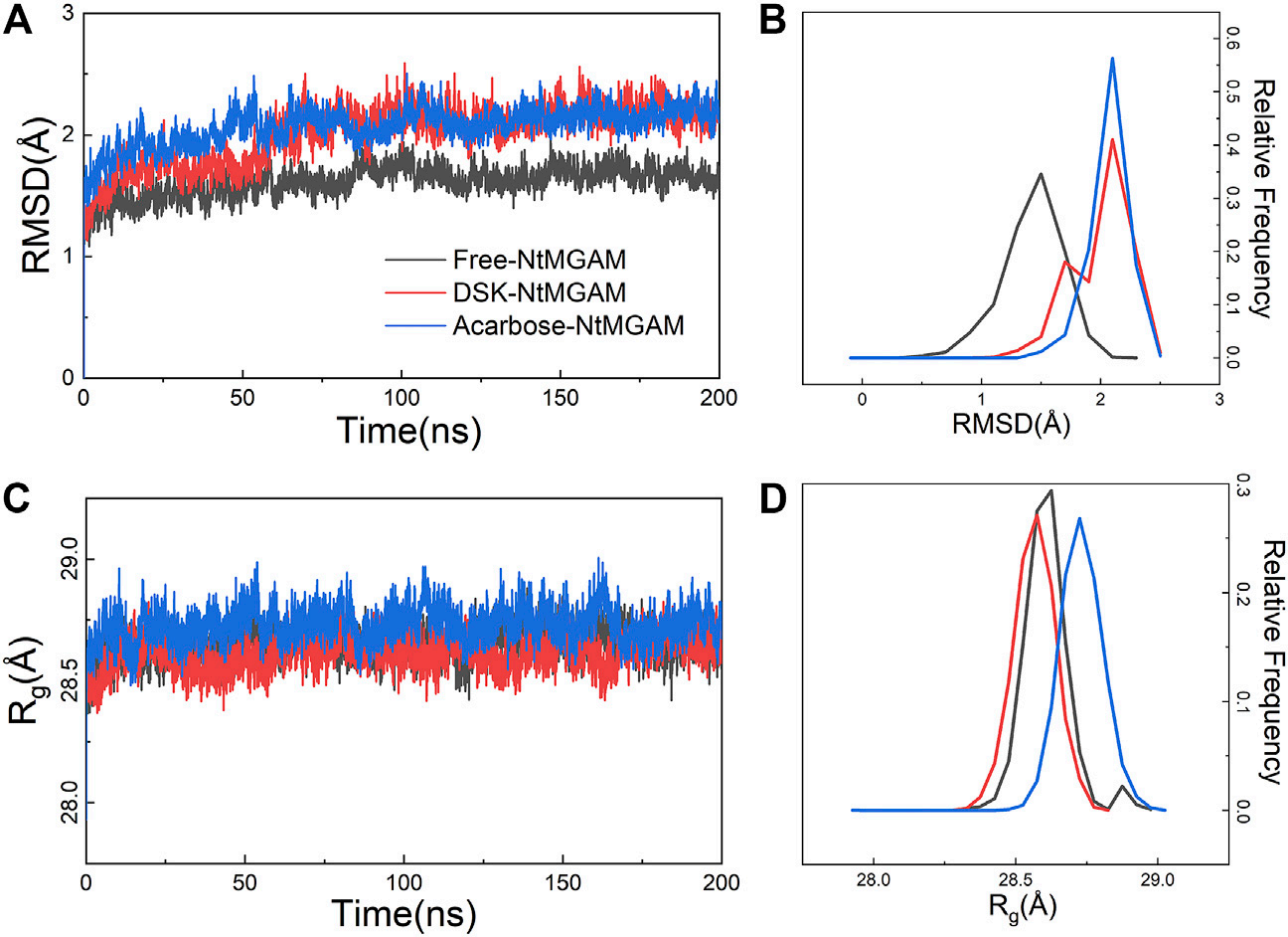
Root-mean-square deviation (RMSD) and radius of gyration (R_g_) analysis of NtMGAM with or without different ligands throughout 200 ns. **(A)** RMSD plot. **(B)** The relative frequency of the RMSD plot. **(C)** Gyration radius (R_g_) plot. **(D)** The relative frequency of the R_g_ plot.

**FIGURE 3:**
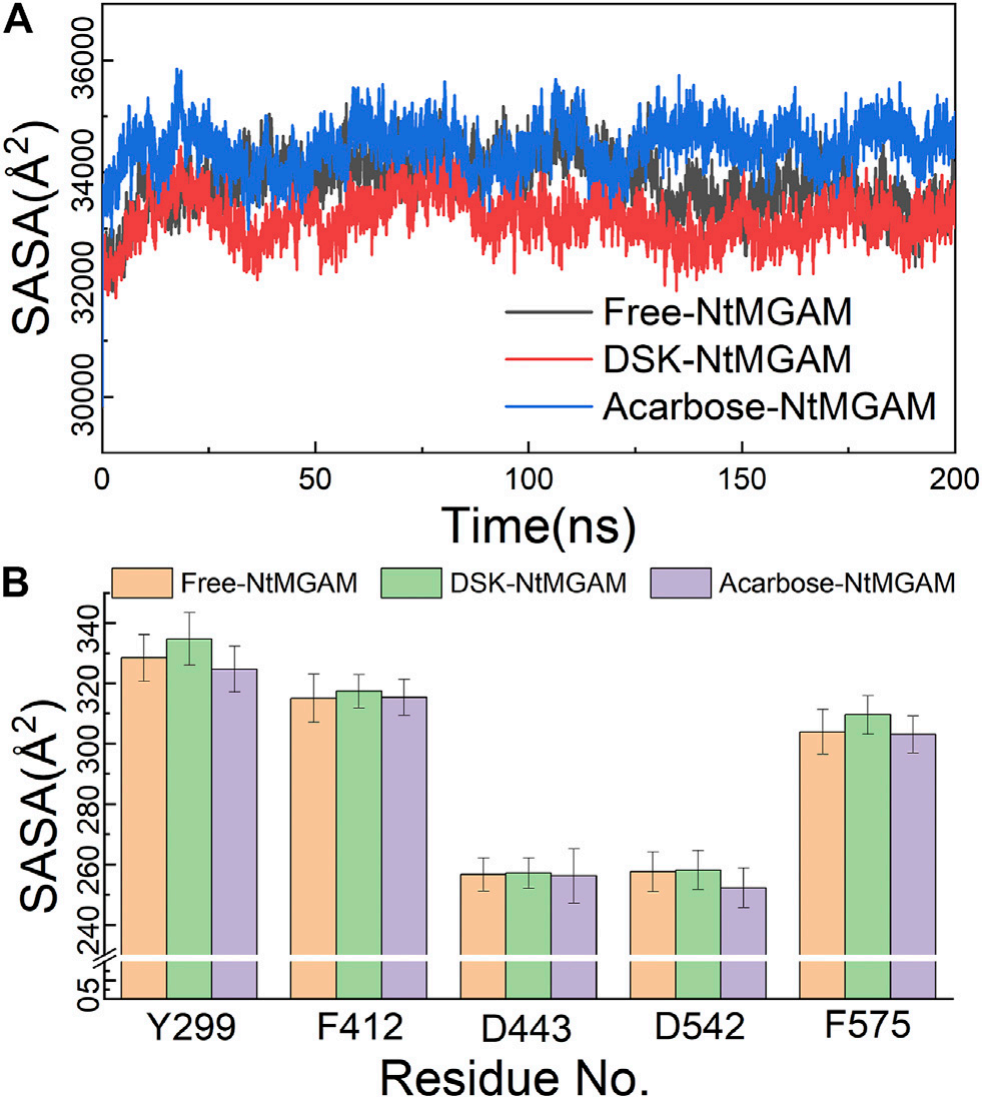
**(A)** Solvent-accessible surface area (SASA) analysis of three systems over 200 ns MD. **(B)** SASA of active residues in different compound structures.

The MD simulations for three systems have been performed three times. The RMSD values of the Cα atom backbone of residues of three systems were calculated to evaluate the equilibrium of systems (Supplementary Figures S3, S4 and Supplementary Table S1). It can be seen in Supplementary Figure S3 that all MD simulations have got equilibrium. In Supplementary Figure S4, the RMSD of the three simulations of free-NtMGAM are slightly different from each other, with the average values of 1.67, 1.94, and 2.13 (from 100 to 200 ns), respectively. Although the RMSD values of each of the three repetitions seldom cross each other, the three simulations have all reached a state of relative equilibrium. The group with the most equilibrious and generally balanced RMSD was chosen for further study (Supplementary Table S1). The parameters of 200 ns MD simulations for three systems were listed in Supplementary Table S2. From Figure 2A, the RMSD values of NtMGAM, DSK-NtMGAM, and acarbose-NtMGAM are stabilized at about 1.67, 2.16, and 2.15 Å, respectively (Figure 2B), suggesting the structures of the three systems had reached a state of relative equilibrium. The R_g_ values of the three systems are shown in Figures 2C,D and are finally stabilized at 28.75 Å. In Figure 3A, the SASA values of acarbose-NtMGAM were stabilized at 34,000 Å^2^; meanwhile, the other two systems were stabilized at 33,000 Å^2^. These results indicate that three systems have attained stability and their states reached equilibrium. SASA values of single residue of the three systems were calculated for core residues. Tyr299, Phe412, Asn433, Asp542, and Phe575 had higher scores in the DSK-NtMGAM complex than the others. It was reported that Asp542 and Phe575 were important residues interacting with the hydroxyl groups of inhibitors binding to NtMGAM (Usman et al., 2019). Our results were consistent with the experimental data. In summary, all the systems were stabilized and can be used for further study.

### Conformational Changes for Inhibitors Binding

**FIGURE 4:**
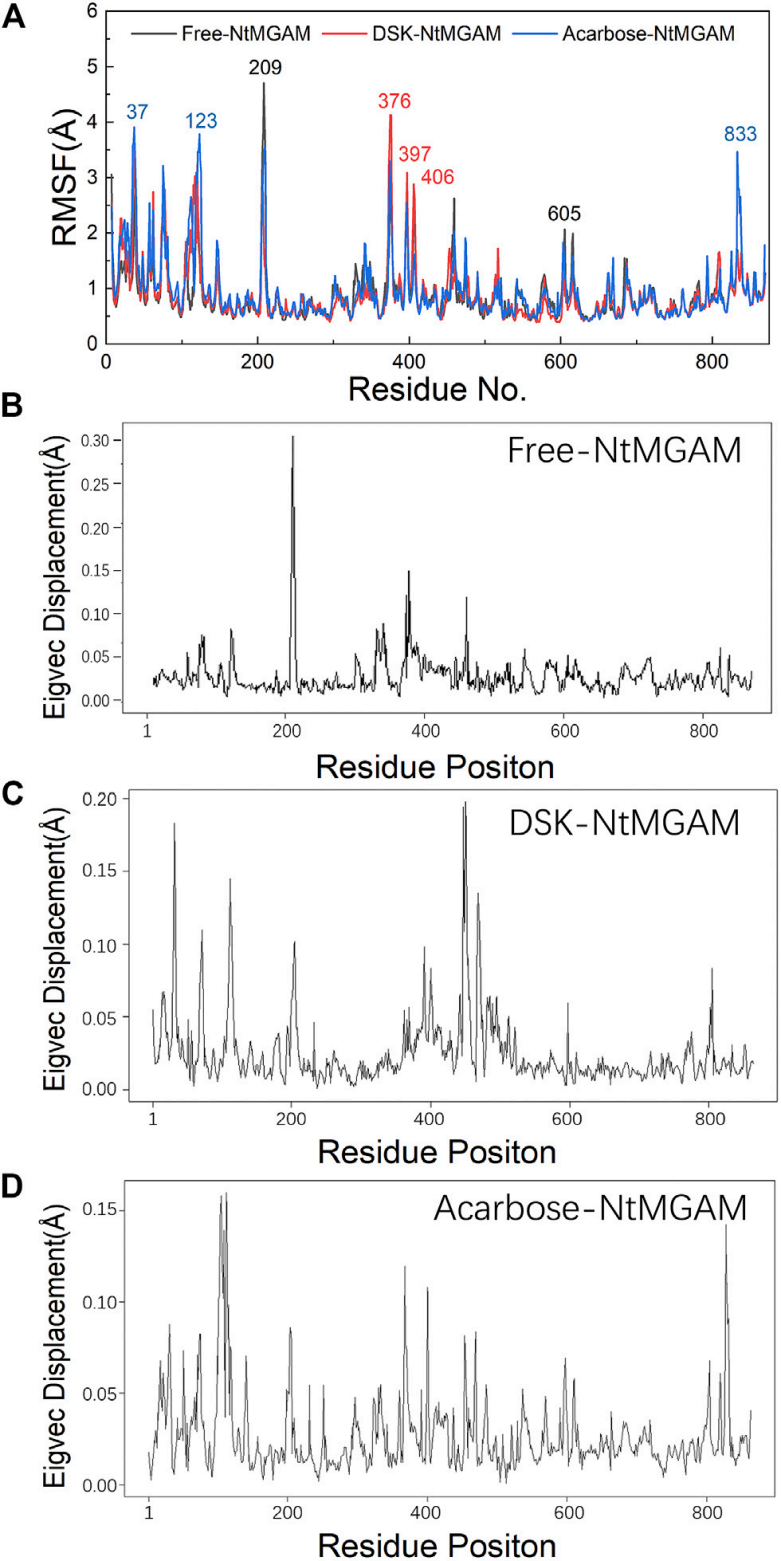
**(A)** Average atom positional root-mean-square fluctuations (RMSF) of the backbone atoms per residue for the inhibitors bound NtMGAM. **(B–D)** The eigenvector components for atomic displacement along the first eigenvectors for MD-generated ensembles of free-NtMGAM, DSK-NtMGAM, and acarbose-NtMGAM, respectively.

**FIGURE 5:**
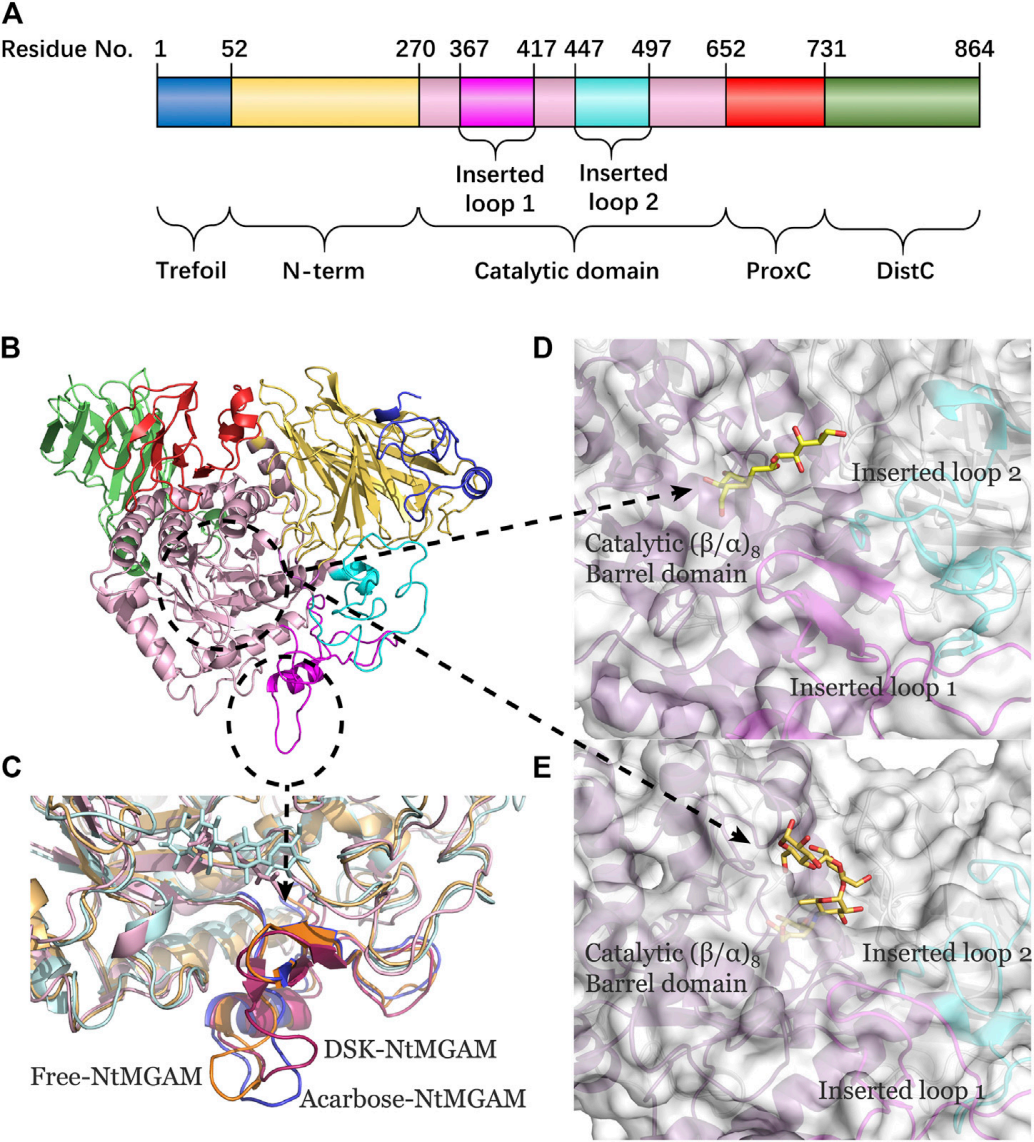
**(A)** The NtMGAM domains with residue numbers labeled. **(B)** New cartoon diagram of NtMGAM with domains colored differently. **(C)** Inserted loop 1 in aligned compounds (the lowest energy conformations from FEL analysis). **(D,E)** NtMGAM active site pocket shown in surface representation occupied by DSK D and acarbose E. The structure of NtMGAM is shown in new cartoon and colored dissimilarly as follows: purple for catalytic domain, pink for inserted loop 1, cyan for inserted loop 2.

To evaluate the deviation amount of displacement in three trajectories from MD simulations, atom positional RMSF values were calculated for the backbone atoms of three systems (Figure 4A). Figures 4B–D show that residue displacements correspond to the motions described by the first eigenvector for three complexes. These displacements represented the relative displacement of each residue caused by the motion described by a given eigenvector. It was reported that NtMGAM contains 868 residues, which can be divided into five structural domains: (I) a trefoil Type-P domain (residue No. 1–51); (II) N-terminal β-sandwich domain (residue No. 52–269); (III) catalytic (β/α)_8_ barrel domain (residue No. 270–651) with two inserted loops [inserted loop 1 (residue No. 367–416) and inserted loop 2 (residue No. 447–492)] protruding out between β3 and α3 and between β4 and α4, respectively; (IV) proximal C-terminal domain (residue No. 652–730); (V) distal C-terminal domain (residue No. 73–868), both with β-sandwich topologies (Sim et al., 2010) (see Figure 5A). In the DSK-NtMGAM complex, residues Ser376, Gly397, and Trp406, which are located at inserted loop 1 domain, exhibited distinct atom positional fluctuation amplitudes. This displacement widened the active site pocket, which would affect the inhibitors' binding.

**FIGURE 6:**
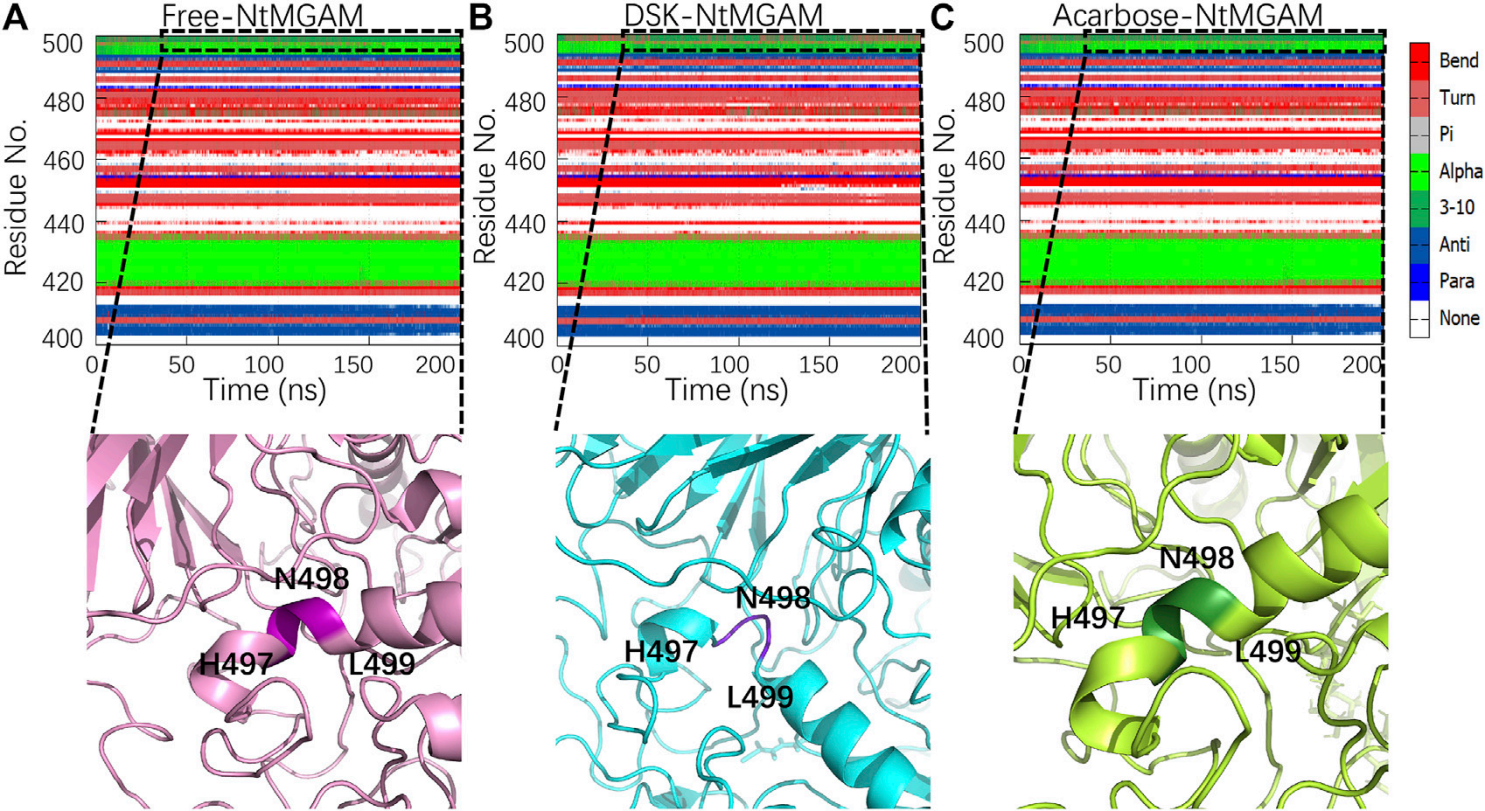
Conformation changes in residues Asn491-Leu493 of the three systems: **(A)** free-NtMGAM, **(B)** DSK-NtMGAM, and **(C)** acarbose-NtMGAM.

Subsequently, secondary structure analysis was also performed, and the corresponding average secondary structure values for each residue are shown in Figures 6A–C and Table 1. It can be seen that the proportion of α-helix in Asn498 in free-NtMGAM and acarbose-NtMGAM was about 90%, whereas in DSK-NtMGAM, it was about 59% (Table 1 and Supplementary Table S3). Supplementary Table S3 shows that the proportion of α-helix in residue 497–499 in free-NtMGAM and acarbose-NtMGAM are almost above 90%. In contrast, in the DSK-NtMGAM complex, the odds are reduced to under 70%. The results revealed that the α-helix of DSK-NtMGAM during 200 ns MD simulations disappeared partly. Residues 497–499 are located near the inserted loop 2 domain, which is quite close to the opening of the (β/α)_8_ barrel. Despiralization of these residues can enlarge the domain of the inserted loop 2, therefore, contributing to the architecture of the inhibitor binding site.

**TABLE 1:** The probability of secondary structures of residues H497 to L499.

Residue	Free-NtMGAM		DSK-NtMGAM		Acarbose-NtMGAM	
	α-Helix	Loop	α-Helix	Loop	α-Helix	Loop
**H497**	0.94	0.06	0.59	0.41	0.89	0.11
**N498**	0.94	0.06	0.59	0.41	0.92	0.08
**L499**	0.94	0.05	0.6	0.40	0.92	0.08

**FIGURE 7:**
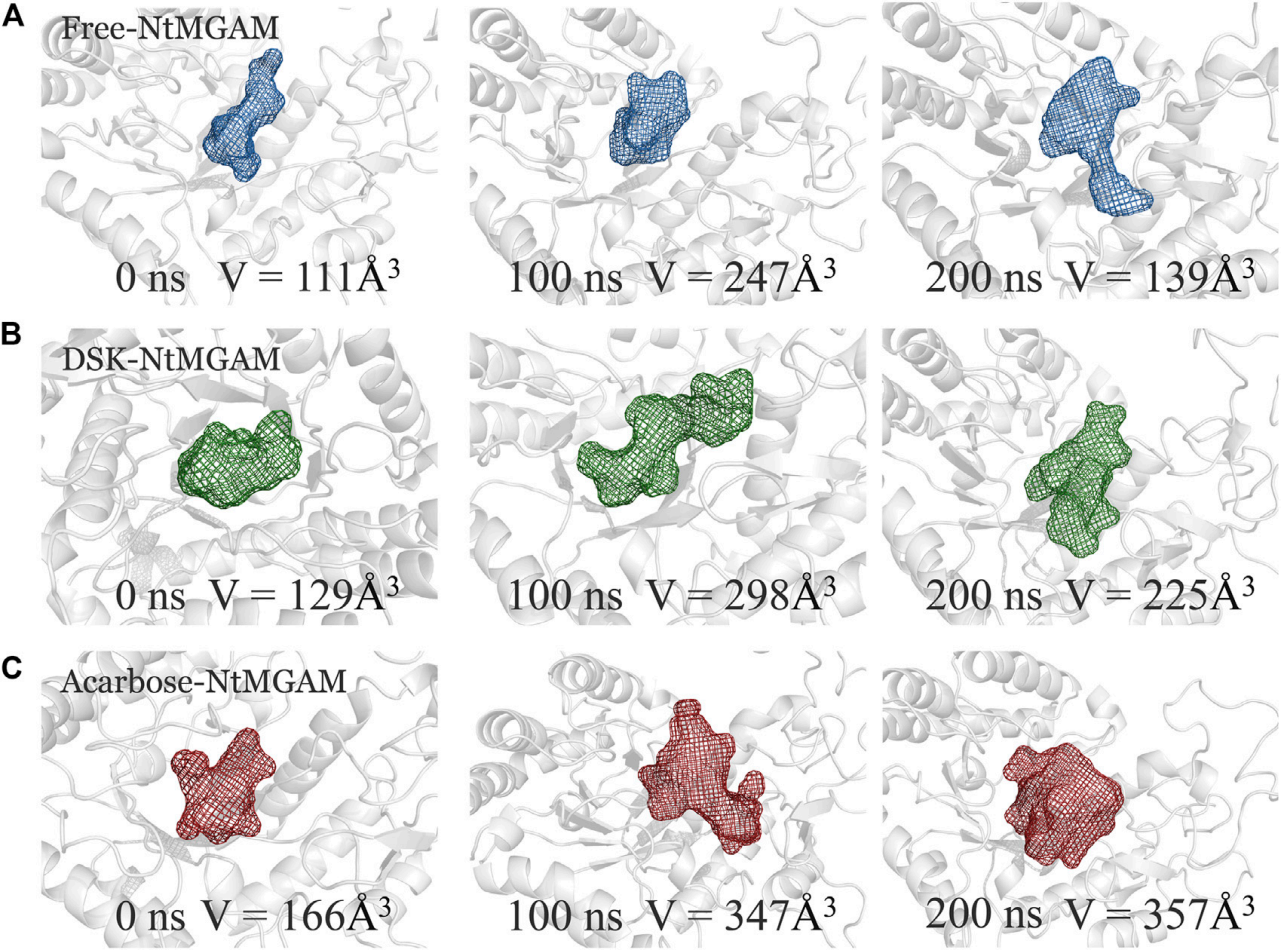
The binding pocket conformations of free-NtMGAM **(A)**, DSK-NtMGAM **(B)**, and acarbose-NtMGAM **(C)** at 0, 100, and 200 ns with the volume of the pockets labeled.

POCASA 44 (http://altair.sci.hokudai.ac.jp/g6/service/pocasa/) (Yu et al., 2010) was utilized to predict the volume of the binding pocket. Parameters are listed as follows: the radius of probe sphere value was 1 Å, single point flag value was 10 Å, and protein depth flag value was 15Å. The active pocket conformations at 0, 100, and 200 ns were shown in Figures 7A–C. The volume of the pocket in free-NtMGAM was smaller than that of DSK-NtMGAM and acarbose-NtMGAM. Obviously, it could be considered that inhibitor of NtMGAM binding to the pocket with the nonreducing sugar ring in the −1 subsite and the reducing ring in the +1 subsite results in net retention of configuration at the anomeric center (Sim et al., 2010). Large active pockets will facilitate the inhibitor binding and entry. However, acarbose is so large that it binds to the NtMGAM active site primarily with its acarvosine unit (−1 and +1 subsite), few with its glycone rings (+2 and +3 subsite) (Sim et al., 2008).

In addition, the S group of DSK can generate charge interaction with residues (Trp400, Asp437, and Asp536) (Supplementary Figure S5), which may stabilize the DSK-NtMGAM complex.

### PCA and FEL Analysis

**FIGURE 8:**
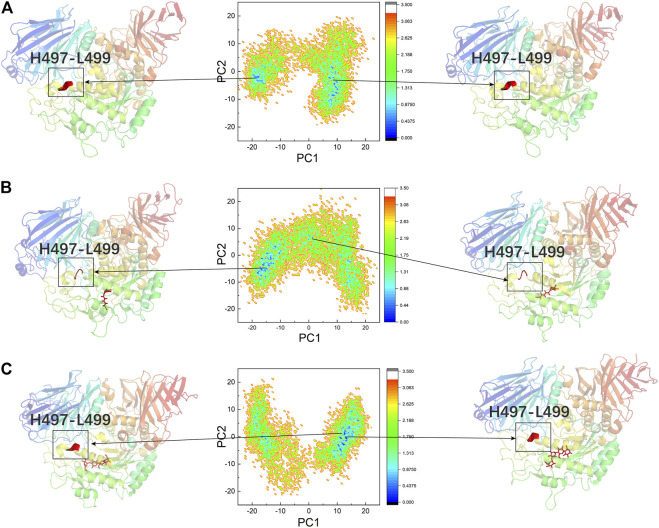
PCA based FEL analysis of NtMGAM **(A)**. DSK-NtMGAM **(B)**. Acarbose-NtMGAM **(C)** as a function of projections of the MD trajectory onto the first (PC1) and second (PC2) eigenvectors. The structures of the two most stable conformations of the three systems are presented with the conformation of Asn491 to Leu493.

PCA was performed to confirm whether the conformational changes of the three systems were continuous and stable, and the results are displayed through FEL (Figures 8A–C). Table 2 lists the PC1 and PC2 probabilities of the three systems. The structures of the most stable conformations of the three systems revealed that the conformational changes in the residues 497–499 domain were complex in DSK-NtMGAM (α-helix disappeared partly). In summary, we can confirm that the conformational changes in the three systems were continuous and stable, and our analyses above are reliable.

**TABLE 2:** The probabilities of PC1 and PC2 of the three systems.

	PC 1 (%)	PC 2 (%)
**Free-NtMGAM**	16.46	6.28
**DSK-NtMGAM**	17.03	6.44
**Acarbose-NtMGAM**	19.61	5.46

### MM-PBSA Calculations

We used the end-point method to calculate the free energy between NtMGAM and the two inhibitors. The results of the Generalized Born (GB) implicit solvent method with a SASA term calculation are shown in Table 3. The binding free energies were mainly contributed by electrostatic energy, which is calculated by the molecular mechanics force field and the electrostatic contribution to the solvation free energy calculated by GB. Meanwhile, the VDW interactions are approximately consistent. As shown in Table 3, the binding free energy of the DSK-NtMGAM complex (−41.90 ± 1.14 kcal/mol) is lower than that of the acarbose-NtMGAM complex (−8.77 ± 1.08 kcal/mol). The results have the same trend as the data calculated from the K_i_ value from Sim’s works (Sim et al., 2008).

**TABLE 3:** MM-PBSA results.

	DSK	Acarbose
**VDWAALS**	−15.84 ± 0.39	−29.86 ± 1.01
**EEL**	−586.14 ± 2.32	−81.12 ± 4.95
**EGB**	565.10 ± 1.55	108.44 ± 4.87
**ESURF**	−5.02 ± 0.06	−6.22 ± 0.19
**ΔG _gas_**	−601.98 ± 2.49	−110.99 ± 4.99
**ΔG _solv_**	560.08 ± 1.50	102.21 ± 4.74
**ΔTOTAL**	−41.90 ± 1.14	−8.77 ± 1.08
**K_i_ (μM)**	0.03 ± 0.01	62 ± 13
**ΔG_exp_**	−10.32	−5.76

### ASMD Simulations

**FIGURE 9:**
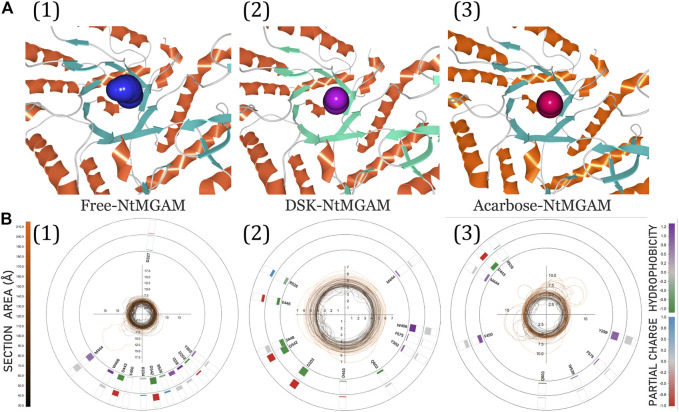
**(A)** 3D visualization of candidate tunnels of (1) free-NtMGAM, (2) DSK-NtMGAM, and (3) acarbose-NtMGAM. **(B)** The details of the tunnel bottleneck contour over time of (1) free-NtMGAM, (2) DSK-NtMGAM, and (3) acarbose-NtMGAM.

The 3D visualization of the channel of the free-NtMGAM, DSK-NtMGAM, and acarbose-NtMGAM obtained by CAVER Analyst 2.0 is listed in Figure 9A. The detailed exploration of the channel bottleneck and surrounding residues is shown in Figure 9B. The bottleneck of the channel in DSK-NtMGAM was larger than that of acarbose-NtMGAM. The surrounding residues displayed around the contour demonstrated the frequency. It can be seen that there are more residues in the DSK-NtMGAM complex comparing to that in the acarbose-NtMGAM complex.

**FIGURE 10:**
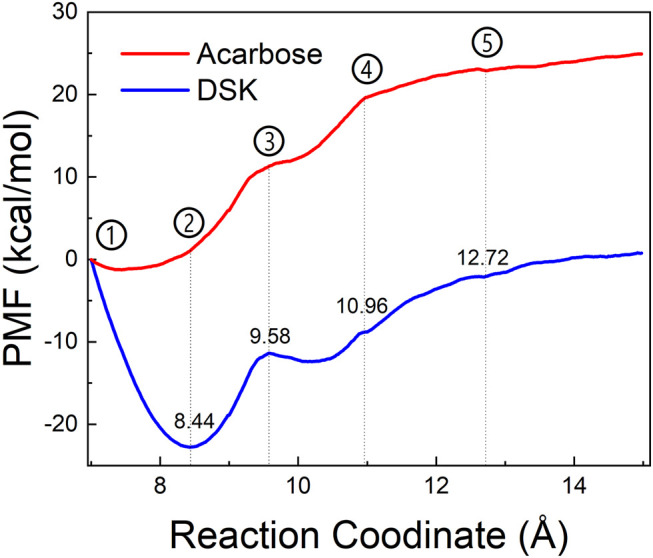
PMF profiles along with the reaction coordinates of acarbose-NtMGAM (red) and DSK-NtMGAM (blue).

To explore the enzyme-inhibitor interactions and the affinity of the active sites of NtMGAM *via* inhibitors unbinding pathway, ASMD simulations were performed on the two complexes. In Figure 10, the PMF profile displayed the energy changes during the process of pulling the ligands out of the NtMGAM channel. In the DSK-NtMGAM complex, a lower energy barrier (13 kcal/mol) should be transferred to completely dissociate DSK from the channel of NtMGAM than that in the acarbose-NtMGAM complex (22 kcal/mol).

**FIGURE 11:**
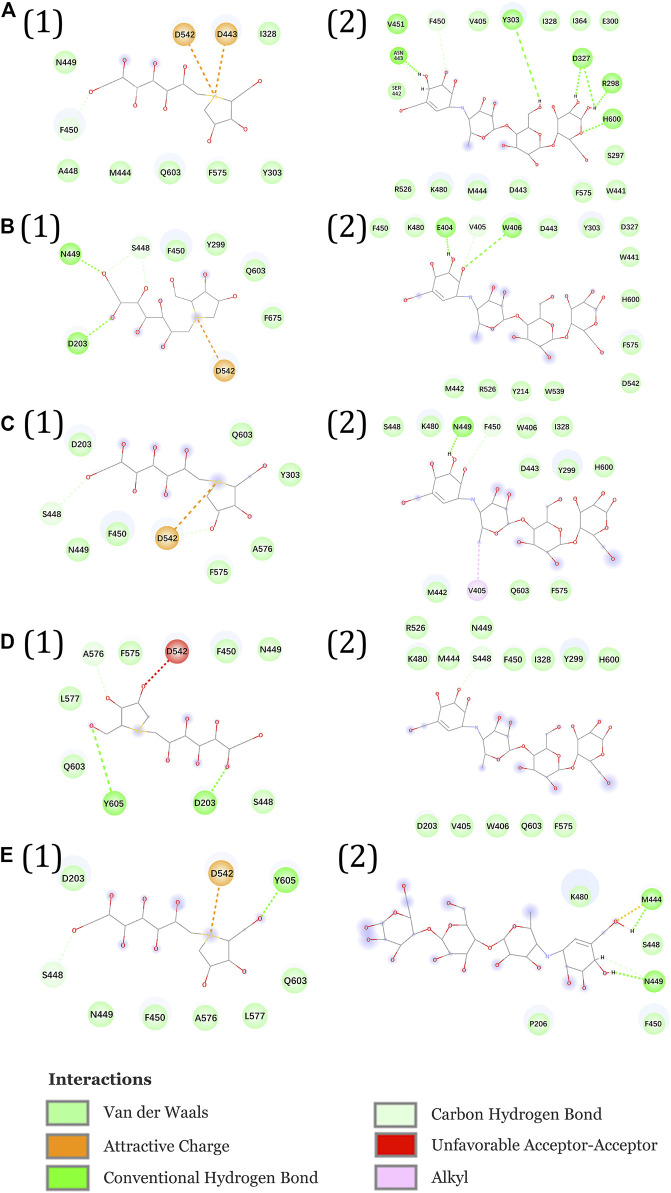
Interactions during ASMD simulations between DSK (1) or acarbose (2) with NtMGAM. **(A)** The initial state of the two systems before the ASMD simulations. Reaction coordinates reached **(B)** 8.44 Å, **(C)** 9.58 Å, **(D)** 10.96 Å, and **(E)** 12.72 Å.

Figures 11A1–E1 show the interactional changes during the dissociation process of DSK-NtMGAM along the reaction coordinate (RC). First, for the initial coordinate (RC = 7 Å) [Figure 11A1], Asp542 and Asp443 formed a salt bridge with DSK. Then, at 8.44 Å [Figure 11B1], the free energy value dropped sharply due to the break of stronger hydrogen bonds between DSK and Asp203 and Asn449 residues. With the movement of DSK, the salt bridge between Asp443 and DSK disappeared and the salt bridge between Asp542 and DSK persisted. Nevertheless, at 9.58 Å [Figure 11C1], the residues that formed hydrogen bonds with the DSK were changed. Except for the salt bridge between Asp542 and DSK, the hydrogen bonds (salt bridge) were disappeared. Thereafter, the interconnections, including hydrogen bonds between channel residues and DSK, increased rapidly after 10.96 Å [Figure 11D1], giving rise to an increase in free energy value. Finally, at 12.72Å [Figure 11D1], DSK completely departed from the channel of NtMGAM and the curve of PMF tended to be flat. Asp542, as an important channel residue, could generate hydrogen bonds with DSK continuously, which was consistent with the results obtained in the channel analysis.

The acarbose dissociating from NtMGAM is shown in Figures 11A2–E2. At the beginning of the ASMD simulation [Figure 11A2], acarbose was tightly fixed due to the strong hydrogen bond interactions with Tyr303, Asp327, Arg298, and His600. Subsequently, at 8.44 Å [Figure 11B2], the free energy value increased slowly because of the stronger hydrogen bond interactions between acarbose and Glu404 and Trp406. At 9.58 Å [Figure 11C2], the acarbose made hydrogen bond interactions with Asn449, Val405, and Phe450, which were stronger than DSK, resulting in the increased free energy value (Figure 11C). Thereafter, the interconnections, including the hydrogen bonds between acarbose and channel residues, disappeared at about 10.96 Å besides the only hydrogen bond with Ser448 [Figure 11D2]. Finally, at 12.72 Å [Figure 11E2], acarbose was completely departed from the unbinding pathways of NtMGAM, and the curves of PMF tended to be flat.

To sum up, compared to acarbose, DSK escaped from NtMGAM easily with lower energy. Asp542 is an important residue on the bottleneck of the active pocket of NtMGAM, which could generate hydrogen bonds with DSK continuously. Our results may provide some useful clues for designing new medicine to relieve symptoms of postprandial hyperglycemia caused by type 2 diabetes. For example, we can modify the 3D structure of acarbose to get a new compound that is suitable for the active pocket of NtMGAM.

## Conclusion

At present, there are multiple drugs for the treatment of type 2 diabetes on the market, including α-glucosidase inhibitors. MGAM has become an efficient drug target for insulin resistance. In order to explore the conformational changes in the active pocket and unbinding pathway for NtMGAM, MD simulations and ASMD simulations were performed between two inhibitors (DSK and acarbose) and NtMGAM. MD simulations indicated that DSK binding to NtMGAM may lead to an enlargement of the active pocket due to the flexibility of the two domains (inserted loop 1 and inserted loop 2), which would facilitate the binding of DSK to NtMGAM. ASMD simulation results indicated that Asp542 was an important channel residue, which could continuously generate hydrogen bonds with DSK. Our results may provide some interesting thoughts for designing new medicine for the treatment of type 2 diabetes based on the molecular structure and specific intermolecular interactions between NtMGAM and DSK substrate in the binding pocket and the entrance channel.

## Data Availability

The original contributions presented in the study are included in the article/Supplementary Material; further inquiries can be directed to the corresponding authors.

## Abbreviations

MGAM, maltase-glucoamylase; NtMGAM, N-terminal human maltase-glucoamylase; CtMGAM, C-terminal luminal subunit; MD, molecular dynamics; DSK, de-O-sulfonated kotalanol; PCA, Principal Component Analysis; PCs, principal components; FEL, free energy landscape; ASMD, adaptive steered molecular dynamics simulations; PMF, potential of mean force; JA, Jarzynski average; RC, reaction coordinate.
