# Supplementary material for: Targeting N-Terminal Human Maltase-Glucoamylase to Unravel Possible Inhibitors Using Molecular Docking, Molecular Dynamics Simulations, and Adaptive Steered Molecular Dynamics Simulations
Source: Front Chem. 2021 Aug 30;9:711242. doi: 10.3389/fchem.2021.711242 (PMC8435576; doi:10.3389/fchem.2021.711242)
Supplement: Supplementary file 1 [file DataSheet2.pdf]

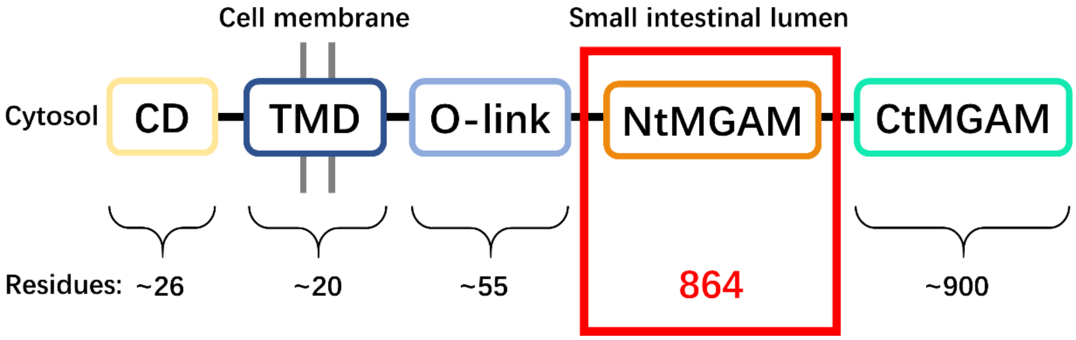

Supplementary Figure 1. MGAM contains following subunits: a small cytosolic domain (CD) containing approximately 26 residues; a transmembrane domain (TMD): 20 residues; an O-glycosylated linker(O-link): 55 residues; and two homologous catalytic subunits: NtMGAM and CtMGAM, each contains less than 900 residues.

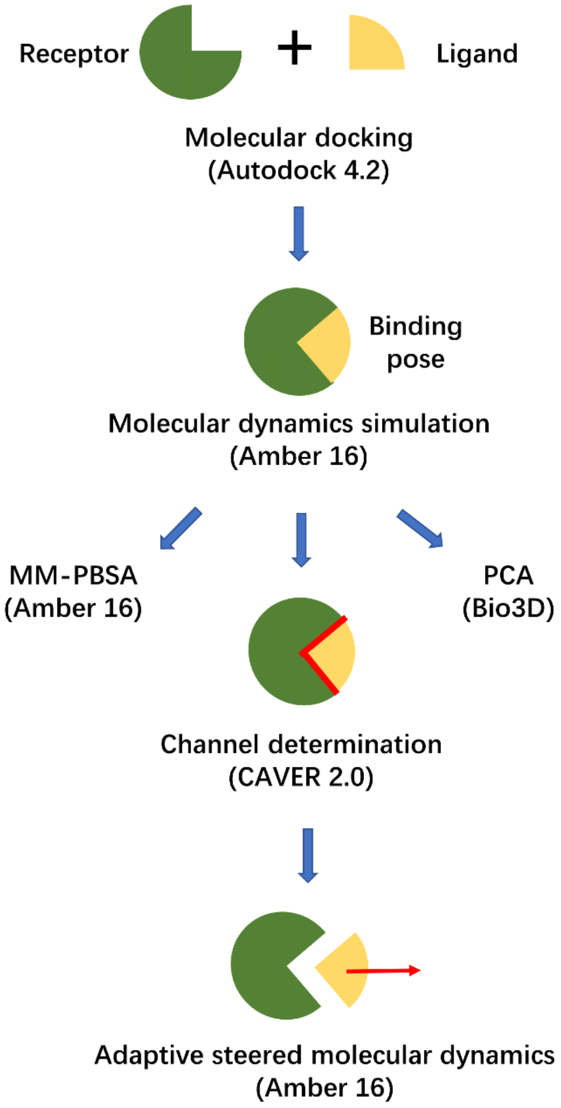

Supplementary Figure 2. Working flow of our study. Firstly, Autodock 4.2 was used to obtain the proper docking pose for acarbose-NtMGAM complex. Secondly, MD simulations were performed on free-NtMGAM, DSK-NtMGAM and acarbose-NtMGAM. Thirdly, MM-PBSA from Amber 16 package was utilized to calculate the free energy for two complexes. Meanwhile, PCA was performed using Bio3d in R to refine structural superposition and to examine the relationship between different conformers. Finally, after defining the proper channel for NtMGAM utilizing CAVER 2.0, ASMD simulations were performed to explore the energy alteration and important channel residues on the bottleneck of active pocket of NtMGAM.

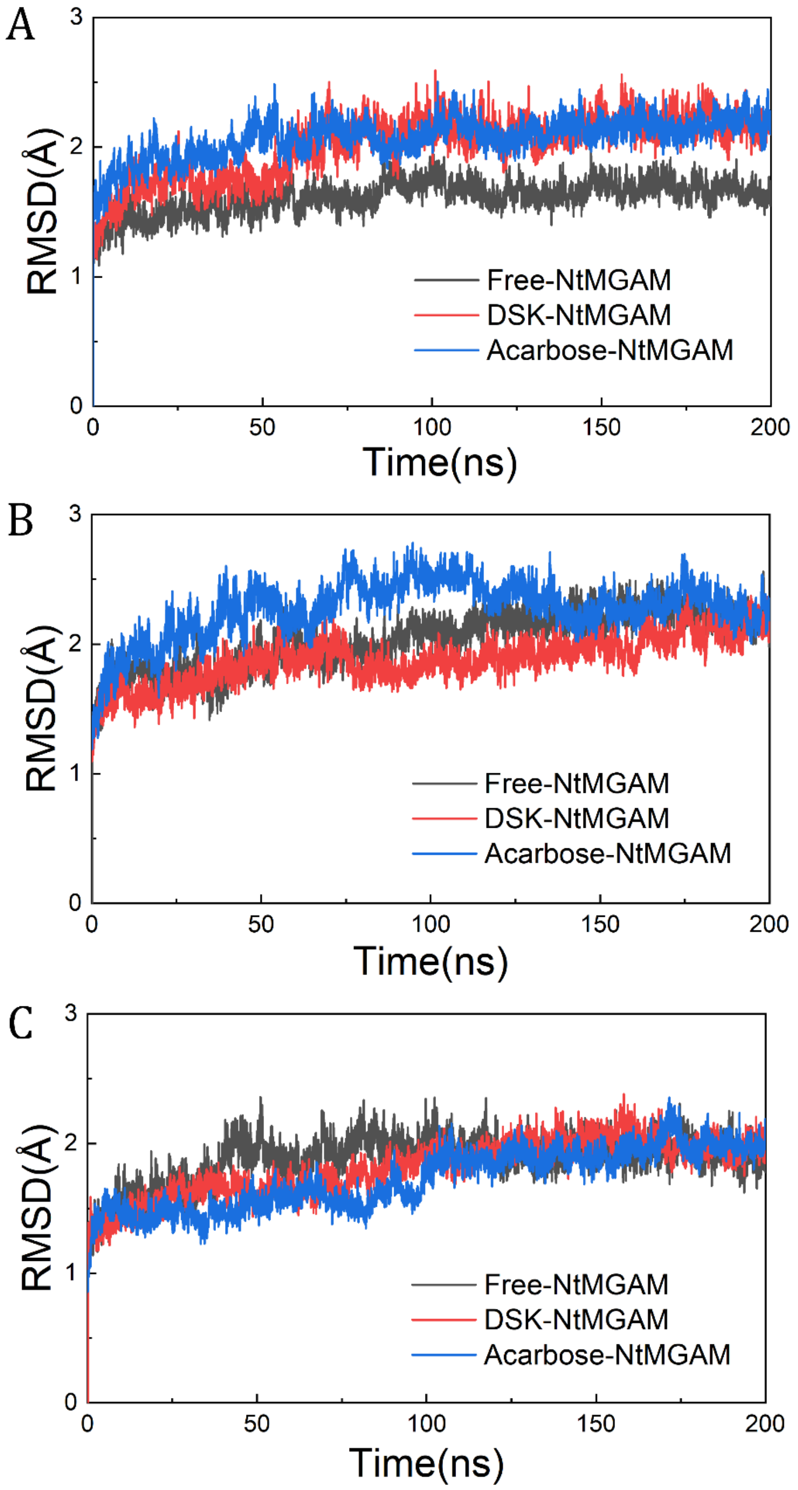

Supplementary Figure 3. RMSD of three systems during 200 ns MD simulations.

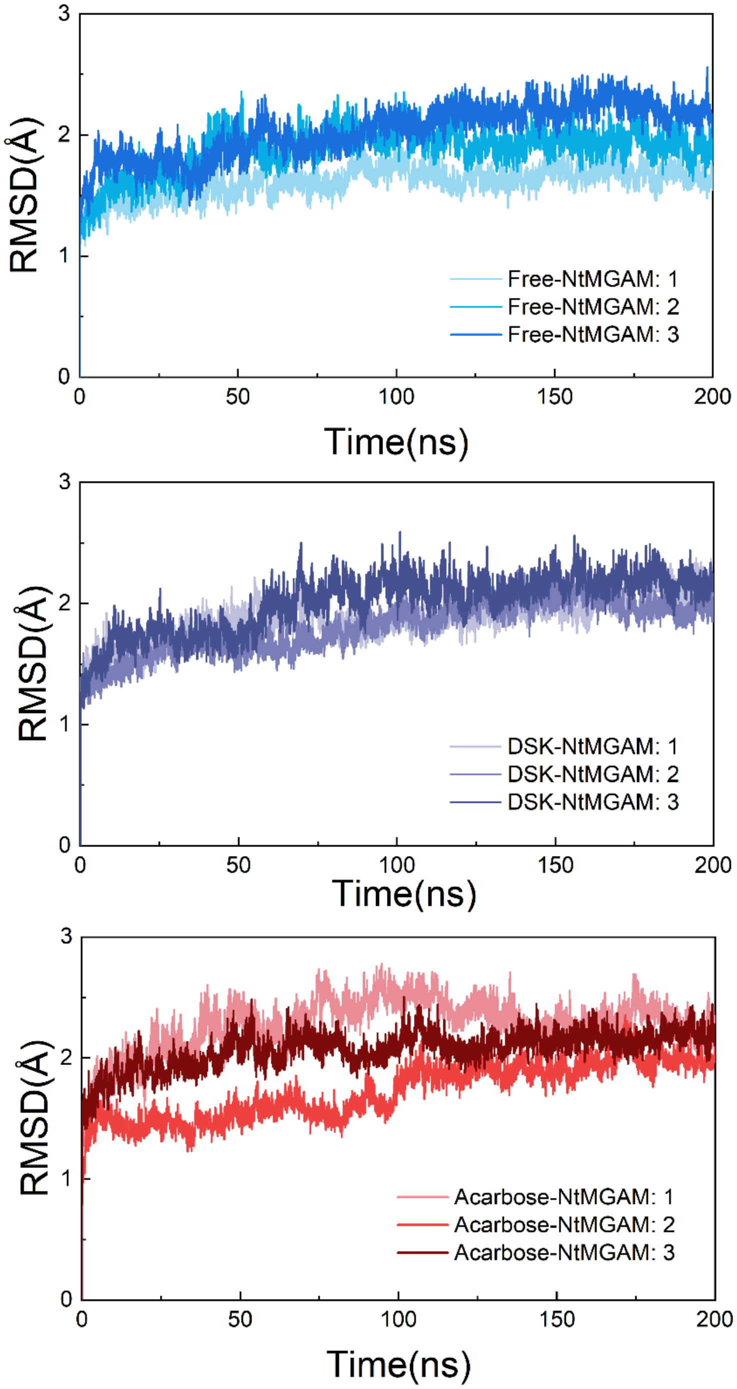

Supplementary Figure 4. RMSD of three repetitions during 200 ns MD simulations.

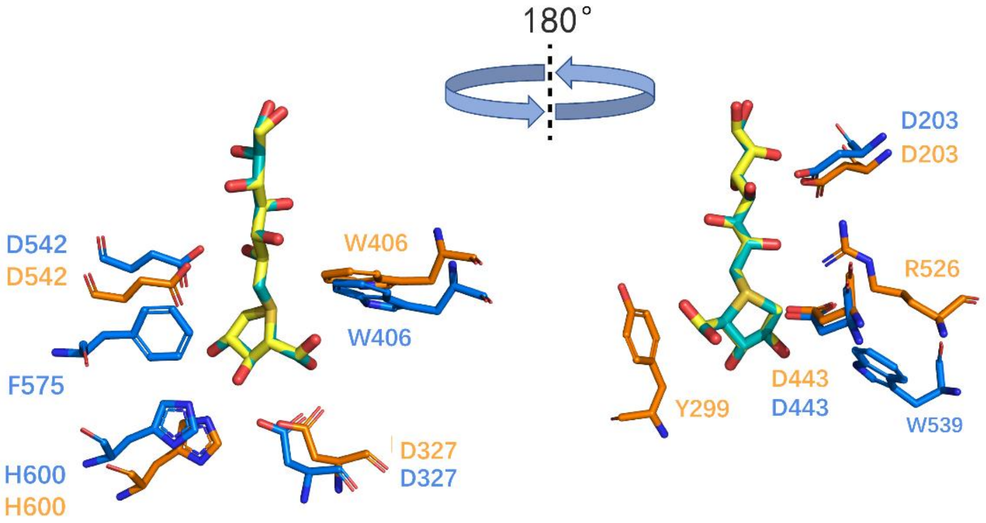

Supplementary Figure 5. Comparison of docking DSK to ntMGAM to the crystal structure 3L4U. DSK is shown as thick sticks in yellow (docking) and cyan (3L4U). The active-site residues are colored in orange (docking) and marine (3L4U) as the residues are labeled in the same colors.

Supplementary Table 1. The average RMSD values of three systems.

	Free-NtMGAM	DSK-NtMGAM	Acarbose-NtMGAM
**100 - 200ns**	1.67	2.16	2.15
**0 - 200ns**	1.61	2.01	2.07

Supplementary Table 2. The parameter of 200 ns MD simulations for three systems were listed.

	Free-NtMGAM	DSK-NtMGAM	Acarbose-NtMGAM
**Total atoms**	28294	28294	27397
**Total molecules**	9141	9138	8825
**Total residues**	864	864	863
**WAT**	8247	8243	7931
**Na^+**	31	30	30

Supplementary Table 3. The probability of secondary structures of residue H497 to L499 in three repetitions.

		Free-NtMGAM		DSK-NtMGAM		Acarbose-NtMGAM	
	**Residue**	**α-helix**	**Loop**	**α-helix**	**Loop**	**α-helix**	**Loop**
**Group 1**	**H497**	0.94	0.06	0.59	0.41	0.89	0.11
	**N498**	0.94	0.06	0.59	0.41	0.92	0.08
	**L499**	0.94	0.05	0.6	0.40	0.92	0.08
**Group 2**	**H497**	0.89	0.10	0.37	0.63	0.93	0.06
	**N498**	0.90	0.10	0.38	0.62	0.93	0.07
	**L499**	0.90	0.10	0.38	0.61	0.93	0.07
**Group 3**	**H497**	0.96	0.04	0.65	0.35	1.00	0.00
	**N498**	0.96	0.04	0.65	0.35	1.00	0.00
	**L499**	0.96	0.04	0.68	0.28	1.00	0.00
